# Assessment of Anthropogenic Pollen Signals in Anatolian Lake Records During the Beyşehir Occupation Phase

**DOI:** 10.3390/plants15111689

**Published:** 2026-05-29

**Authors:** Hülya Caner, Gülan Güngör

**Affiliations:** 1 Department of Marine Geology and Geophysics, Institute of Marine Sciences and Management, Istanbul University, 34000 Istanbul, Türkiye; 2 Institute of Social Sciences, Istanbul University, 34000 Istanbul, Türkiye; gulangungor3636@gmail.com

**Keywords:** pollen data, anthropogenic indicators, multivariate analysis, PCA, Anatolia, Late Holocene

## Abstract

Understanding the extent to which anthropogenic activity shapes vegetation dynamics is a central challenge in palaeoecology. In the Eastern Mediterranean, pollen-based studies have traditionally identified human impact through qualitative interpretations of anthropogenic indicators, particularly within the framework of the Beyşehir Occupation Phase (BOP). However, proxy-based quantitative comparison of anthropogenic signals across multiple sites remains limited. This study compiles pollen datasets from multiple lacustrine records across Anatolia (Türkiye) to construct a regional multi-site dataset and evaluates anthropogenic influence using a quantitative BOP period anthropogenic taxa integrated with Principal Component Analysis (PCA). The relative representation of pollen indicators enabling the determination of anthropogenic effect was evaluated using a composite pollen index based on *Olea*, *Juglans*, *Plantago lanceolata*-type, *Cerealia* and *Rumex acetosa*-type taxa. The results reveal substantial spatial variability in anthropogenic signals, with combined pollen percentages ranging from less than 1% to 16% among lakes. PCA results show clear inter-site differentiation, with the first two components explaining 42.94% and 21.95% of the total variance, respectively. In particular, *Olea* emerges as the most influential indicator, strongly contributing to the primary ecological gradient. These findings provide a proxy-based quantitative extension of the traditionally qualitative BOP concept and show that selected anthropogenic pollen indicators are spatially heterogeneous across Anatolian lake records. By integrating a composite anthropogenic index with multivariate analysis, this study offers a robust and transferable framework for comparing human–environment interactions across different regions and ecological settings.

## 1. Introduction

Understanding the timing, intensity, and spatial variability of anthropogenic impacts on vegetation is a central objective in palaeoecology and environmental archeology. Pollen-based reconstructions provide one of the most robust tools for tracing past human environment interactions, as they directly record vegetation responses to both climatic and anthropogenic drivers [1,2,3]. In the Mediterranean and Near Eastern regions, particular attention has been given to anthropogenic pollen indicators, including cultivated taxa and disturbance-related species, which reflect agricultural expansion, arboriculture, and land use intensification.

Regional studies from Türkiye further highlight the importance of local environmental and cultural contexts in modulating anthropogenic signals. Palynological investigations from central and western Anatolia have demonstrated significant variability in vegetation response to human activity during the Late Holocene [4,5,6,7]. These studies emphasize that anthropogenic indicators are strongly influenced by regional ecological settings and cannot be interpreted uniformly across different landscapes.

Comparable anthropogenic signals have also been documented across Europe, particularly in Mediterranean and temperate regions [2,8,9]. These records often show synchronous increases in cultivated taxa and secondary anthropogenic indicators during key historical periods such as the Roman era, the Medieval Climate Anomaly, and later phases of intensified land use. However, the expression of these signals varies across regions due to differences in ecological settings, land use strategies, and cultural trajectories.

Despite these advances, direct comparison of anthropogenic signals across regions remains challenging. Pollen records are inherently influenced by local environmental conditions, including basin characteristics, sedimentation processes, and vegetation composition, which may obscure regional-scale patterns. Furthermore, the BOP concept has traditionally been applied qualitatively, based on visual interpretation of pollen diagrams, limiting its comparability across multiple datasets.

Within this framework, the BOP has emerged as a key concept for identifying periods of intensified human activity in Anatolia. First recognized in lacustrine pollen records from southwestern Türkiye [10,11], and further developed through integrated palaeoecological and archeological studies [12,13,14], the BOP is characterized by increases in cultivated taxa such as *Cerealia*, *Olea* and *Juglans*, together with disturbance indicators including *Plantago lanceolata*-type and *Rumex acetosa*-type. These pollen assemblages are widely interpreted as reflecting agricultural intensification, arboriculture, and broader socio-economic transformations during the Late Holocene. This period was named the Beyşehir Occupation Phase based on the pollen record from the Beyşehir settlement, where the anthropogenic signal is clearly observed [9,10,11,12]. During this period, evidence of forest clearance, crop cultivation such as cereals and arable weeds, and tree cultivation/arboriculture activities such as walnut, olive, manna ash and vine is observed. Although the chronological boundaries of the Beyşehir Occupation Phase may vary depending on study areas and the age models of pollen records, the literature indicates that this phase generally began around 3500 cal. BP and continued, at least in some places, until approximately 1300 cal. BP. At the end of the BOP, regrowth of pine appears to have occurred in woodlands where pine became dominant; however, in pollen diagrams before this period, pine was often found together with oak, juniper or cedar [9,12]. In Eastern Anatolia, the presence of human impact during the broadly contemporaneous Late Holocene period appears different from the traces of the classic BOP period identified in southwestern Anatolia.

To overcome these limitations, recent studies have increasingly adopted multivariate statistical approaches, particularly PCA, to identify dominant gradients in pollen datasets and to disentangle anthropogenic signals from natural variability [15]. However, few studies have explicitly tested whether BOP-type signals are embedded within the dominant structure of multivariate variability or whether they remain dependent on site-specific interpretations.

This study evaluates the relative representation and compositional structure of selected human-derived pollen indicators in various lake records across Anatolia by defining an index based on a composite indicator. Through standardization of pollen variables from selected taxa, both the relative level and compositional pattern of anthropogenic impact across lake systems are examined comparatively. In this context, the study presents a semi-quantitative extension of human-induced pollen signalling, which has traditionally relied largely on qualitative interpretations, and provides a robust analytical framework for comparing human–environment interactions in different ecological and geographical contexts.

## 2. Materials and Methods

**Figure 1:**
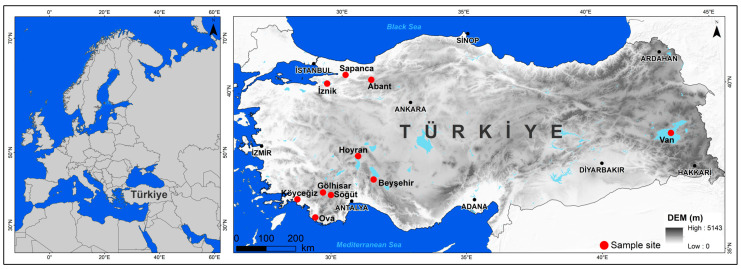
Location of the studied lacustrine records across Anatolia (Türkiye), including İznik, Abant, Sapanca, Söğüt, Beyşehir, Hoyran, Gölhisar, Köyceğiz, Ova and Lake Van (VAN1 and VAN2). The sites cover different climatic and ecological regions, providing a spatial framework for evaluating vegetation dynamics and anthropogenic signals.

Pollen datasets from Abant, Sapanca, Beyşehir, Hoyran, Gölhisar, Köyceğiz, Söğüt, Ova, and Van (VAN1 and VAN2) lakes across Anatolia were compiled from the Neotoma Paleoecology Database to create a regional multi-site dataset; site locations are shown in Figure 1 [10,11,13,16,17,18,19,20]. Such synthesis approaches are increasingly used to investigate large-scale vegetation dynamics and human environment interactions [9,21].

Restricting the dataset to Neotoma records ensures a reproducible and methodologically consistent regional synthesis, consistent with recent approaches that emphasize standardized datasets for large-scale vegetation and human environment analyses. The original publications corresponding to each pollen record, together with their Neotoma dataset identifiers and chronology sources, are provided in Table 1.

The analysis relied on five taxa that could directly and indirectly represent human impact: *Olea*, *Juglans*, *Plantago lanceolata*-type, *Cerealia* and *Rumex acetosa*-type. These taxa were considered suitable indicators for assessing traces of anthropogenic signals in pollen records, as they could be associated with agricultural activity, cultivated trees, open field use, weed environments, and grazing pressure [2,10,12,23]. The selection of these taxa is based on their consistent representation across all records examined and their use in previous studies. While additional taxa such as *Castanea*, *Fraxinus ornus*, *Pistacia* and *Platanus* have been used elsewhere, their presence in the compiled dataset was inconsistent and generally limited to a small number of regions. Inclusion of such taxa would reduce the comparability of the multi-region dataset and increase the rate of missing values, thus weakening the robustness of the composite index and subsequent multivariate analyses. Therefore, the selected taxa represent a compromise between ecological representativeness and statistical consistency, ensuring that the resulting anthropogenic signal is both regionally comparable and methodologically robust. Only directly related pollen names were considered in taxon matching. For example, *Olea* was included exclusively at the genus level, while broader family-level categories such as Oleaceae were excluded. Similarly, the *Rumex* variant was limited to only *Rumex acetosa* and *Rumex acetosa*-type records, not to the general genus level. In the first stage, rows corresponding to selected anthropogenic indicator taxa from 1300 to 3500 cal. BP were identified in each lake record, and pollen counts of these taxa were collected to calculate a composite anthropogenic indicator value. This value was divided by the total pollen count during the BOP period to obtain a composite anthropogenic pollen percentage representing the relative share of the five selected taxa within the total pollen. The relative presence of selected anthropogenically derived pollen indicators was assessed using the combined pollen percentage obtained from the five selected indicator taxa. In this study, *Olea*, *Juglans*, *Plantago lanceolata*-type, *Cerealia* and *Rumex acetosa*-type were aggregated and expressed as a percentage of total pollen. These taxa represent agricultural activity, grazing pressure, and arboriculture, and are widely used as indicators of human impact in Mediterranean pollen records [23,24]. The temporal resolution and continuity of pollen records from selected lakes in the 1300–3500 cal. BP range differ. Samples included in the analysis were determined by considering the published age–depth model and chronological framework of each record. Therefore, a one-to-one simultaneous sample-based comparison across all lakes is not possible in the dataset. Instead, the study presents an integrated lake-based comparison of selected anthropogenic pollen markers within a broadly defined BOP time interval. Sample density, temporal resolution, and possible chronological gaps between selected pollen records can affect how the anthropogenic signal is represented. Therefore, the results do not show the continuous change in human impact over time in the 1300–3500 cal. BP range but rather compare the relative levels and compositional differences in anthropogenic pollen markers among lakes within this period.

In the second stage, the minimum, maximum, arithmetic mean, and standard deviation of the combined anthropogenic pollen percentage were calculated for each lake in the 1300–3500 cal. BP range. Thus, not only the overall lake-based representation of the five selected taxa but also the variability they exhibited throughout the 1300–3500 cal. BP period was evaluated.

The five selected anthropogenic indicator taxa were converted into percentage values for each lake separately; thus, a lake and taxon ratio matrix was created where the percentages of the lakes were in the rows, and the percentages of the taxa were in the columns. This matrix was used to evaluate which taxon combinations originated from the combined anthropogenic pollen signal and to provide the basic data structure for multivariate analyses such as a heatmap and PCA. To reduce the effects of differing variances among taxa and to standardize the dataset prior to multivariate analysis, all variables were transformed using z-score normalization (z = (x − μ)/σ), where x represents the pollen percentage, μ is the mean, and σ is the standard deviation of each variable [25]. In the final stage, PCA was applied to standardized taxon percentages. In the analysis, observations represent the lakes, and variables represent the standardized percentages of five selected taxa. The aim of PCA is to summarize the similarities and differences between the lakes using a smaller number of components and to reveal the compositional patterns of anthropogenic indicators.

PCA was applied to the standardized dataset to identify the main gradients in pollen assemblages and to explore relationships between vegetation dynamics and anthropogenic indicators. PCA was performed on the covariance matrix of z-score standardized variables, including all selected terrestrial pollen taxa. This method is widely used in palaeoecology to reduce dimensionality and identify dominant ecological gradients [25,26]. The first two principal components (PC1 and PC2), explaining the largest proportion of variance, were retained for interpretation.

## 3. Results

When five anthropogenic indicator taxa were selected in the 1300–3500 cal. BP time intervals were evaluated together, and significant differences were found between the lakes in terms of both total signal level and taxonomic composition. The combined anthropogenic pollen percentage calculated on a lake-by-lake basis reveals the share of these five taxa in the total pollen during the relevant time interval and allows for a comparative evaluation between different records. In this respect, the highest combined value was found in İznik (16.7%) and the lowest value in Hoyran (0.9%). İznik Lake is followed by Ova (7.9%), Van 2 (5.5%), Van 1 (4.5%), Söğüt (4.2%) and Gölhisar (3.9%). Beyşehir (3.0%), Sapanca (1.3%), Abant (1.7%) and Köyceğiz (1.3%) have lower combined anthropogenic pollen percentages. This distribution clearly shows that the selected anthropogenic indicators are not represented with the same intensity in all lakes, and therefore the reflection of human influence in pollen records varies on a regional scale (Table 2).

The mean of the combined percentages throughout the BOP period also supports this pattern. In terms of mean value, İznik (16.6%) ranks first, followed by Van 2 (5.7%), Ova (4.7%), Van 1 (4.5%), Söğüt (4.2%), Gölhisar (3.6%) and Beyşehir (2.8%). Lakes with lower mean values are Abant (2.0%), Sapanca (1.5%), Köyceğiz (1.2%) and Hoyran (0.9%). These results show that the lakes can be evaluated at three different intensity levels in general. İznik, Van 2, Ova, Van 1 and Söğüt stand out as records with mean combined pollen percentages of approximately 4% and above, where the selected indicators are more strongly represented. Abant, Sapanca, Köyceğiz and Hoyran, with mean values below approximately 2%, emerged as lakes with weaker anthropogenic indicators. Gölhisar and Beyşehir are in the middle group, between these two groups (Table 2).

Maximum values are important for understanding the extent to which the selected indicators are concentrated. The fact that the maximum value in Iznik reaches 36.9% indicates the presence of very strong anthropogenic signals in this record during the BOP period. The maximum value of 25.4% in Ova is similarly noteworthy. Maximum values are 13.9% in Söğüt, 9% in Van 2, 7% in Van 1, 7% in Beyşehir, 8.6% in Gölhisar, 8.1% in Abant, 4.8% in Sapanca, 2,9% in Köyceğiz and 2.5% in Hoyran (Table 2). The fact that Iznik and Ova stand out compared to other records in terms of both total percentage and maximum value suggests that human influence produced a stronger or more visible pollen signal in these lakes.

**Figure 2:**
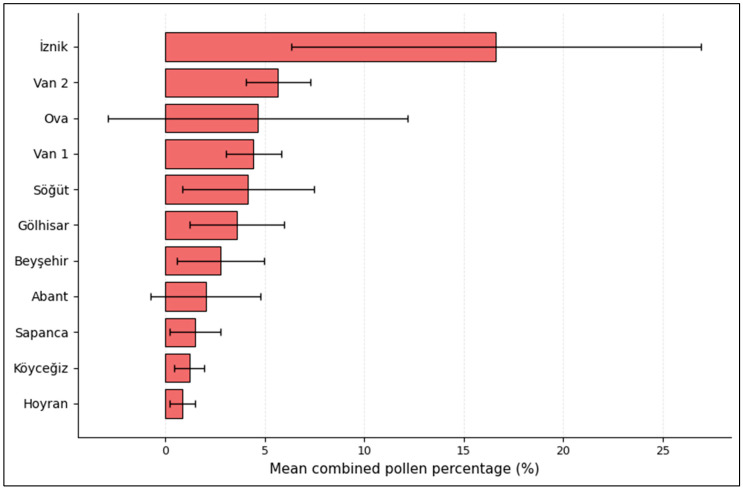
Lake-based average values and standard deviations of combined pollen percentage for selected anthropogenic indicator taxa in the 1300–3500 cal. BP range. Lakes are ranked according to average combined pollen percentage, and error bars indicate the standard deviation.

The temporal variability of total pollen percentage also differs among the lakes, and the standard deviation calculated from the total percentage series reveals how much this signal fluctuates over time. The standard deviation is 10.3 in İznik, 7.5 in Ova, 3.3 in Söğüt, 2.8 in Abant, 2.4 in Gölhisar, 1.6 in Van 2, 2.2 in Beyşehir, 1.4 in Van 1, 1.3 in Sapanca, 0.7 in Köyceğiz and 0.7 in Hoyran (Table 2). This distribution shows that İznik and Ova not only have high total percentages but also exhibit highly variable records over time. In contrast, lakes such as Hoyran and Köyceğiz have both low overall anthropogenic pollen levels and exhibit more limited fluctuations over time. A high standard deviation indicates that the selected indicators exhibit a more variable pattern, strengthening and weakening over specific periods, while a low standard deviation suggests a weaker but relatively stable human influence (Figure 2).

**Figure 3:**
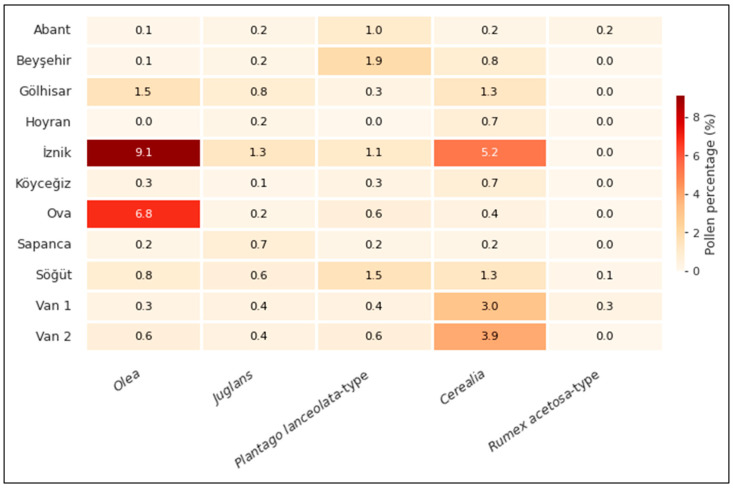
Heatmap showing the percentage distribution of selected anthropogenic indicator taxa in lakes, spanning the period of 1300–3500 cal. BP.

The heatmap showing the percentage distribution of selected anthropogenic indicator taxa at the lake level reveals that the combined pollen signal differs not only quantitatively but also in terms of taxonomic composition (Figure 3). İznik stands out particularly with its high values for *Olea* and *Cerealia*; *Juglans* and *Plantago lanceolata*-type also contribute secondarily to this lake. *Olea* is largely dominant in the plain, while *Cerealia* and *Juglans* are less represented. In the Van 1 and Van 2 records, the anthropogenic signal shows a more *Cerealia-dominant* distribution. In Söğüt, the closer values of *Plantago lanceolata*-type, *Cerealia*, *Olea* and *Juglans* indicate a more balanced composition where multiple indicators contribute together. In Gölhisar, *Olea* and *Cerealia* are dominant. In contrast, the *Plantago lanceolata*-type has become relatively more dominant in Beyşehir, Abant and partly in Sapanca, suggesting that the anthropogenic signal in these lakes may be more closely related to open land use and ruderal environmental indicators. In Köyceğiz and Hoyran, both the total signal is low and none of the selected taxa show significant dominance. Therefore, the heatmap clearly shows that the differences between the lakes are due not only to the total intensity of the selected anthropogenic indicators but also to which combinations of taxa generate this signal.

**Figure 4:**
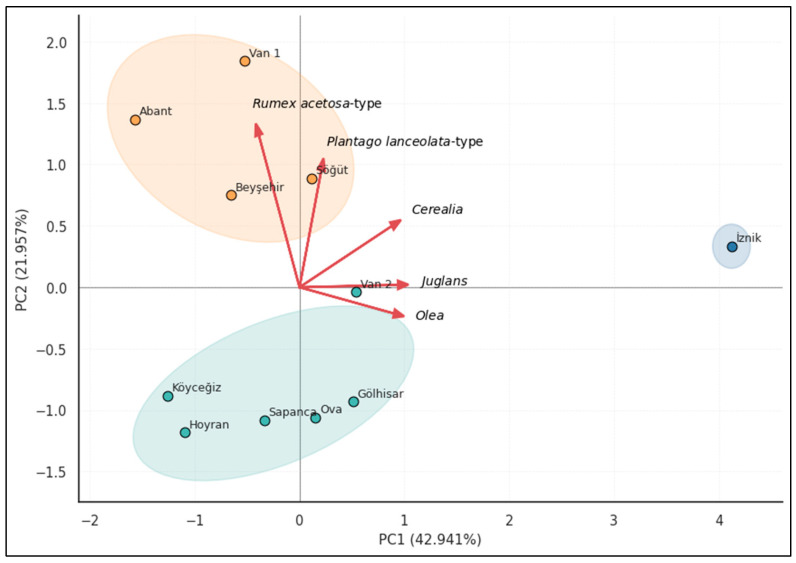
Lake-based PCA results of selected anthropogenic indicator taxa.

PCA, performed after z-score standardization, shows that the selected anthropogenic indicator taxa differ between lakes not only in terms of total density but also in terms of pattern (Figure 4). The first two components explain approximately 64.89% of the total variance; 42.94% is represented by the first component and 21.95% by the second. The first component stands out as an axis particularly associated with *Olea*, *Juglans* and to a lesser extent *Cerealia*, while the second component appears to be more associated with *Plantago lanceolata*-type and *Rumex acetosa*-type. This suggests that PC1 more strongly reflects the anthropogenic signal associated with cultivated trees and agricultural activity, while PC2 more strongly reflects the impact associated with open field use, ruderal environments, and potential grazing pressure. The PCA results show three distinct clusters among the lakes. First, İznik stands apart from all other records, positioned alone on the positive side of PC1. This indicates that the selected anthropogenic signal is particularly strong in the Olea and Juglans axis in İznik, suggesting that indicators related to cultivated trees and agricultural activities are more dominant in this record. Second, Ova, Gölhisar, Sapanca, Köyceğiz and Hoyran cluster more towards the negative PC2 side; the structure of the selected taxa in these lakes deviates from the *Plantago lanceolata*-type and *Rumex acetosa*-type dominant pattern on the upper axis. Third, Abant, Beyşehir, Söğüt, and Van 1 are more located towards the positive PC2 side, exhibiting a distribution closer to the *Plantago lanceolata*-type and *Rumex acetosa*-type orientation. The separation of Van 2 from Van 1 in the PCA ranking stems from a methodological artefact arising from z-score standardization. The complete absence of the *Rumex acetosa*-type species in the Van 2 record creates a normally magnified difference between the two records. Ecologically, both Van records share a *Cerealia-dominant* anthropogenic signal consistent with the high-altitude continental environment of Eastern Anatolia, and this artefact does not affect the interpretations.

In conclusion, the selected anthropogenic indicators do not show a uniform effect in the lake records of Anatolia. Accordingly, in some lakes, the anthropogenic effect is represented more by indicators related to cultivated trees and grain production, while in others it is represented by indicators related to open land use, ruderal environments, and grazing pressure (Figure 4).

## 4. Discussion

The results of this study demonstrate that anthropogenic signals are systematically embedded within the dominant gradients of pollen variability across Anatolia. This finding aligns with a growing body of evidence indicating that Late Holocene vegetation dynamics in the Eastern Mediterranean cannot be explained by climatic forcing alone but are strongly shaped by human activities [3,8,9]. Regional syntheses have shown that agricultural expansion, the development of arboriculture, and increasing land use intensity produced persistent and spatially structured anthropogenic signals in pollen records. In particular, the co-occurrence of tree-crop indicators (e.g., *Olea*, *Juglans*) with disturbance taxa is widely regarded as a robust proxy for human-induced landscape transformation [27].

The separation of lake records along the PCA axes reflects both regional environmental differences and varying degrees of anthropogenic influence. The strong differentiation of İznik along PC1, coupled with its high and highly variable BOP period values, suggests that this record captures pronounced and dynamic human environment interactions. In contrast, the low and stable BOP period values observed in Hoyran indicate relatively weak or more stable anthropogenic influence. This spatial heterogeneity highlights the importance of local ecological and socio-economic contexts in modulating human impact. Similar patterns have been documented in Mediterranean and European pollen records, where anthropogenic indicators show regionally variable expressions depending on land use intensity, settlement dynamics, and environmental constraints [8,9]. This supports the interpretation that the distribution of Late Holocene vegetation in Anatolia was shaped by a combination of climate and human-induced processes, and that human influence acted as a strong and local variable controlling the environment.

The combined anthropogenic pollen index applied in this study represents a significant methodological advancement over traditional qualitative approaches. By integrating multiple indicators *Olea*, *Juglans*, *Plantago lanceolata*-type, *Cerealia* and *Rumex acetosa*-type into a single quantitative framework, the method captures multiple dimensions of land use, including cultivation, grazing, and disturbance. This reduces reliance on individual taxa and increases the robustness of anthropogenic signal detection. Moreover, the standardized structure of the index enables direct comparison across sites, facilitating regional synthesis. Its integration with multivariate analyses such as PCA allows anthropogenic signals to be evaluated within the broader structure of ecological variability, providing a more rigorous assessment of their relative importance. The percentages of pollen obtained from different lakes are influenced by factors such as lake basin size, presence or absence of streams, topographic structure, basin morphology, sedimentation conditions, pollen source area, and total pollen count. These factors can affect the representation and comparability of individual pollen taxa across regions [3]. However, since this study relies on published open pollen datasets with different sampling strategies and basin characteristics, these variables could not be fully controlled in the current multi-region synthesis. Therefore, the composite anthropogenic pollen index should not be interpreted as an absolute measure of anthropogenic influence. Instead, it is used as a comparative indicator of the relative representation and taxonomic composition of selected anthropogenic pollen markers in each lake’s pollen record. Thus, the results obtained are interpreted in conjunction with each lake’s local geographic, ecological, and basin-specific context. Additionally, applying z-score normalization to selected anthropogenic taxa prior to PCA helps to reduce the dominance of absolute abundance differences between taxa by standardizing each variable according to its mean and variance, but does not eliminate watershed-specific effects.

*Olea europaea* is a significant indicator of human-induced land use, but its appearance depends on the geographical and ecological context. Olive (*Olea europaea* L.) is considered the most prominent and economically important fruit tree in the Mediterranean Basin, providing edible fruit and storable oil [27]. In countries bordering the Mediterranean, olive groves constitute a significant component of food production. In habitats characterized by a Mediterranean climate, olive trees (*Olea europaea* L. subsp. *europaea var. sylvestris* (Mill) Lehr) grow as part of evergreen plant communities within garrigue and maquis vegetation, usually in hilly areas [28]. Wild olive is considered a sensitive biological indicator for the Mediterranean bioclimatic region [29]. In contrast, olive cultivation has led to the species exceeding its natural bioclimatic limits, resulting in the cultivation of domesticated olives (*Olea europaea* subsp. *europaea var. sativa*) at higher altitudes and latitudes, as well as in drier areas than their natural habitats [27]. In this study, the use of *Olea* as an anthropogenic indicator is not based on a reinterpretation of this taxon, but rather on its established interpretation in the BOP literature. Records from the Mediterranean and sub-Mediterranean regions reveal that *Olea* is generally associated with olive cultivation, tree cultivation, and human land use, especially in conjunction with other anthropogenic indicators. Therefore, in this study, *Olea* is included in the composite anthropogenic pollen index in accordance with the existing interpretation and classification in the relevant literature. At the same time, the geographical and ecological limits of *Olea* are considered in the interpretation, and its presence is not considered a uniform indicator of olive cultivation in all Anatolian records.

Anthropogenic pollen signals detected in selected lake records in Anatolia should be interpreted not only in terms of their intensity but also in relation to locally specific land use strategies, ecological constraints, and socio-economic conditions. The BOP was initially defined as a period in southwestern Anatolia characterized by indicators such as deforestation, agriculture, grazing-related degradation, and the cultivation of cereals, weeds, and fruit trees such as walnuts, olives, manna, and vines [10,12,23]. This suggests that the classical BOP signal in southwestern Anatolia originates not from a single anthropogenic activity but from a mixed land use system involving agricultural practices. In the present study, İznik has the highest total anthropogenic pollen percentage and stands out particularly with the strong representation of *Olea*, as well as *Cerealia* and *Juglans*. Lake İznik is in a transitional position between different climatic zones, namely Mediterranean and Black Sea climates, and vegetation zones, namely European–Siberian and Mediterranean floristic zones of the Marmara region and has a long settlement history. Lake İznik’s low altitude (85 m), temperate sub-Mediterranean climate, hot summers, and sufficient rainfall are suitable for the ecological needs of *Olea europaea* in northwest Anatolia, and it allows for sustainable and productive olive cultivation both during the BOP period and still to this day. Miebach et al. [16] reported that evidence of human activity in the Lake İznik basin is more pronounced from the Early Bronze Age onwards, with the emergence of fruit trees, crops, and secondary anthropogenic indicator taxa, and that the changes between deforestation and subsequent agricultural use and vegetation renewal become more pronounced. Therefore, the İznik signal can be associated with a landscape where tree cultivation, cereal cultivation, and forest clearing are carried out together in an ecologically favourable northwestern Anatolian environment [16]. Furthermore, Lake İznik is located right next to ancient Nicaea, one of the most strategically and economically important cities of the Eastern Roman Empire. The presence of dense urban populations, extensive roads for the empire, and agriculture is the reason for the most intense anthropogenic influence observed among all the study areas. In Lake İznik’s records, elevated *Olea* values coincide with reduced forest taxa, indicating large-scale vegetation clearance and the expansion of olive-based agroecosystems [16]. The magnitude and persistence of these increases exceed natural expectations, pointing to deliberate cultivation rather than climatic expansion. Importantly, *Olea* occurs in association with other anthropogenic indicators, reflecting integrated land use systems rather than isolated taxonomic fluctuations. At a regional scale, olive pollen is consistently linked to long-term landscape transformation and the intensification of tree-crop economies [3,27]. This role is directly reflected in the PCA results. The alignment of *Olea*-rich assemblages with PC1 indicates that arboriculture-driven land use constitutes a primary driver of the dominant ecological gradient. This confirms that anthropogenic processes are embedded within the core structure of vegetation variability and supports the interpretation of PC1 as a human-driven gradient consistent with BOP dynamics.

Although the human impact signal associated with BOP appears more limited in the study by Eastwood et al. [12] at Lake Ova, this difference is likely largely due to chronological placement. A later revised age–depth model by Giesecke et al. [22] shows that the increase in *Olea* in Lake Ova record corresponds to approximately 3177 cal. BP, thus presenting a signal more consistent with BOP. The results obtained in this study also reveal that anthropogenic impact is recorded at Lake Ova, and that this signal is primarily represented by *Olea*. Lake Ova, located on the Mediterranean coast of southern Anatolia, almost at sea level, reflects a similar but compositionally different pattern. In the pollen record, olive is almost alone in the anthropogenic taxon composition, while other human-derived taxa account for less than 1%. This indicates that the anthropogenic pattern in this record is largely *Olea* dominant. The region’s classic Mediterranean climate, with hot–dry summers and mild rainy winters, is more suitable for olive cultivation than grain cultivation. The ancient city of Patara, located near the lake, has a settlement history dating back to 5500 BC, and its position in the Hellenistic and Roman maritime trade networks may have made olive cultivation prominent in terms of economic gain.

Lake Köyceğiz is perhaps the most ecologically complex example, and despite being located near sea level on the southwest Aegean coast, in a climate ideal for olive cultivation, it exhibits one of the lowest total anthropogenic pollen percentages among all regions. Despite its proximity to the Mediterranean coast, Lake Köyceğiz does not show strong traces of agricultural activities such as olive cultivation, which would be expected to correspond to the Hellenistic–Roman period in pollen records [10,12]. The fact that the total percentage of the five anthropogenic indicators selected in this study is 1.3%, one of the lowest levels among the lakes, shows that this weak representation cannot be explained solely by chronological inconsistencies, as in Lake Ova. Therefore, it can be said that the anthropogenic signal associated with BOP in the Köyceğiz record is either quite weak or expressed in a limited way compared to other regional records. This complex situation can be explained by the lake system’s unusual basin morphology. The presence of relatively rugged and steep terrain to the east of the lake limits the area of arable flat land in the basin, while the presence of wetlands and wetland vegetation between the lake and the delta may have suppressed terrestrial agricultural pollen contributions. Lake Köyceğiz is a coastal lagoon lake system near the sea, connected to the sea by a canal. The limited flat agricultural hinterland and the dominance of coastal and wetland vegetation in the basin may structurally suppress human-induced pollen contributions independently of anthropogenic activities. Furthermore, archeological evidence from the ancient Kaunos site located in the Köyceğiz basin indicates that the economy was primarily a maritime economy focused on fishing rather than large-scale agriculture [30].

Similarly, the fact that the total proportion of the five selected anthropogenic indicator taxa in Lake Hoyran remains at a very low level of 0.9% is consistent with Eastwood et al. [12] assessment that this record does not reflect a clear BOP signal. Located in a semi-arid basin at approximately 920 m above sea level, Hoyran’s climate and altitude fall outside the optimum range for olive cultivation. The relatively stable low standard deviation of the anthropogenic signal indicates that human pressure was not only weak but also temporally consistent, pointing to a low intensity and stable land use process. Hoyran Lake appears to have remained relatively sparsely populated and agriculturally natural throughout the BOP period. Its remote location from the sea, its distance from major trade routes, and its relatively limited agricultural potential may have prevented the dense settlement and land use concentration observed in more accessible or ecologically favourable areas.

Lake Söğüt exhibits a balanced multi-taxon composition with closely related contributions from the taxa *Olea*, *Juglans*, *Plantago lanceolata*-type and *Cerealia*. This diverse anthropogenic signal may reflect a mixed land use pattern developing at different elevation zones, rather than a uniform land use pattern. The location of Lake Söğüt in southwestern Anatolia at approximately 1400 m elevation limits the interpretation of olive cultivation as a direct anthropogenic activity around the lake. Therefore, the *Olea* signal may be related to the influence of olive pollen from lower altitude areas with a Mediterranean climate, while the *Cerealia*, *Plantago lanceolata*-type and *Juglans* signals may be related to cereal farming, pasture use and tree cultivation carried out on higher slopes or around the lake basin. This suggests that the Söğüt record presents a composite pollen signal where different production methods coexisted on a regional scale during the BOP period.

The moderate *Olea* and *Cerealia* signals in Gölhisar Lake may similarly reflect a dual land use pattern. However, Gölhisar’s location at approximately 950 m altitude makes it a more favourable location for olive cultivation. Therefore, the *Olea* signal should be evaluated not directly as widespread olive farming, but in conjunction with local topographic protection, favourable microclimate conditions, or pollen contributions from lower altitude surrounding areas. In contrast, the presence of *Cerealia* may be reflected in the pollen records of cereal farming in or near the catchment areas around the lake. In this respect, Gölhisar is an intermediate example where olive and cereal production are represented together, but at a limited intensity.

In Lake Beyşehir, the dominance of the *Plantago lanceolata*-type over cultivated tree taxa indicates that the anthropogenic signal is primarily influenced by open field degradation, grazing pressure, and pasture use. Its location in a basin with semi-arid and continental characteristics, approximately 1120 m above sea level, means it remains above the reliable ecological threshold, especially for olive cultivation. Therefore, the anthropogenic signal in the Beyşehir record appears to be related more to agricultural pasture economics, open field use, and pastoral activities than to intensive olive-based BOP characteristics [2,12].

Located in northwestern Anatolia at an altitude of approximately 1350 m above sea level, Lake Abant is situated in a humid and temperate forest region ecologically unsuitable for olive cultivation. The dominant signal of *Plantago lanceolata*-type in this region may be related to pastoral land use in forested areas with limited agricultural potential. Despite its geographically favourable, low altitude location in Northern Anatolia, Lake Sapanca also gives a weak anthropogenic signal. Both lakes have a more humid and rainier climate compared to other lakes. Although closer to Lake İznik, Sapanca’s climatic conditions are variable. The lake basin may have diluted pollen contributions from spatially limited agricultural areas; furthermore, the dense forest cover of its basin may have restricted agricultural expansion regardless of settlement density.

The Van 1 and Van 2 records indicate that during the Late Holocene, approximately concurrently with the BOP, Eastern Anatolia produced an anthropogenic signal different from the classic BOP model of Southwestern Anatolia. In this study, the anthropogenic signal in the Van records is not predominantly *Olea* but is primarily represented by the *Cerealia* taxon. Due to its continental climate conditions, steppe vegetation, and high-altitude of 1650 m, the Lake Van basin was not ecologically suitable for Mediterranean-like land use based on agricultural tree cultivation. Therefore, a signal of BOP based on olive cultivation should not be expected in Van records. Anthropogenic activities around Lake Van became evident 3800 years ago, when the increase in *Plantago lanceolata* pollen indicated disturbance caused by livestock Wick et al. [31]. However, indications that agriculture played an important role in the region are weak, because wild cereals and other Gramineae species producing cereal-type pollen, together with weeds such as *Polygonum aviculare*, *Papaver rhoeas* and *Mercurialis annua*, have occurred naturally in the steppes of the Near East since the Late Glacial and are therefore not suitable indicators of agricultural fields. However, archaeobotanical data provide more direct evidence for the presence of agricultural production around Van. In the study by Dönmez and Belli [32], plant remains were obtained from Yoncatepe, a Urartian settlement located in Van province and dated to the Iron Age (1st millennium BCE). In the storage rooms of the Yoncatepe palace, large quantities of hulled barley (*Hordeum vulgare* L.) and bread/durum wheat (*Triticum aestivum* L./T. durum Desf.) were found mixed with small amounts of domesticated emmer wheat (*Triticum dicoccum* Schübl.). These findings indicate the existence of an agricultural economy based especially on cereal production around Van during the Iron Age. Therefore, the anthropogenic signal in the Van records reflects not the characteristics of a BOP dominated by olive cultivation, but a different socio-ecological structure associated with cereal farming in steppe vegetation conditions dependent on a continental climate.

The spatial differences in anthropogenic pollen signals identified in this study are not unexpected, as previous paleoecological studies from Anatolia have shown that the characteristics of the BOP vary considerably between regions. The regional differences observed here are largely consistent with the synthesis study presented by Woodbridge et al. [9], which shows that Holocene vegetation dynamics and the intensity of human activity in southern Anatolia are spatially heterogeneous and strongly influenced by regional ecological and cultural conditions [9]. Their work also showed that anthropogenic indicators associated with tree cultivation, agricultural activity, and landscape opening vary considerably between regions and over time, reflecting differences in settlement history, land use intensity, and environmental conditions [9]. This study supports this broader regional interpretation while demonstrating that such variability can also be expressed quantitatively through differences in the compositional structure and relative intensity of selected anthropogenic pollen indicators across the BOP range. The distinct distinction between lake records in the PCA results demonstrates that anthropogenic signals in Anatolia are not represented by a uniform land use pattern but rather reflect regionally specific socio-ecological systems. In this context, the novelty of the present study lies in not only recognizing spatial variability but also demonstrating it within a standardized, multi-regional, and semi-quantitative analytical framework. By integrating selected anthropogenic pollen indicators into a composite index and evaluating their compositional relationships via PCA, the study shows that regional variability is linked not only to the intensity of anthropogenic influence but also to the differences in land use structure represented in each lake record.

Miebach et al. [16] determined in their study that the tree cultivation signal associated with BOP in Lake Iznik plays a secondary role when compared with southwestern Anatolian records. This study presents a different picture by applying a standardized composite index simultaneously to eleven records. Lake Iznik contains the highest proportion of composite anthropogenic pollen among all regions (16.7%), a result that could not have been determined without systematic multi-region quantitative comparison. This suggests that the intensity of anthropogenic signals in Anatolian lake records can be underestimated in areas where they are generated by combinations of various taxa rather than a single taxon; it shows that quantitative multi-region approaches can reveal patterns that qualitative or single-region methods might miss. Indeed, Woodbridge et al. [9], in their pollen records from 21 regions in Southwest Anatolia, found that the OJCV (olive, walnut, chestnut, and grape) index in Lake Ova showed the earliest increase among all the records examined; however, they evaluated this finding only in the context of the chronological spread of fruit tree cultivation in Anatolia and did not quantitatively compare the signal intensity with other areas. On the other hand, the present study shows that Lake Ova ranks second among the areas examined in terms of composite anthropogenic signal intensity (7.9%), indicating that the anthropogenic pollen signal is strong in BOP.

Comparison with European records reveals both broad consistencies and important regional contrasts in the expression of anthropogenic signals. While increases in anthropogenic indicators are widely associated with land use intensification and landscape opening across Europe, the stronger variability observed in Anatolia likely reflects its pronounced ecological heterogeneity and its position at the interface of Mediterranean and continental climatic systems [8]. This transitional setting enhances the sensitivity of vegetation to both climatic fluctuations and human pressure, resulting in more spatially heterogeneous and site-specific expressions of anthropogenic impact. Consequently, Anatolian pollen records do not simply replicate broader European trends but instead highlight the context-dependent nature of human environment interactions, where local environmental constraints and socio-economic trajectories play a decisive role. The patterns identified in this study are also consistent with broader regional and supra-regional syntheses across the Eastern Mediterranean and Europe. Increases in cultivated taxa such as *Olea* and *Juglans*, coupled with changes in vegetation structure, have been widely associated with intensified human activity and land use expansion [8,18]. At a more regional scale, high-resolution records from the Dead Sea region further demonstrate that vegetation dynamics during the Late Holocene reflect the combined influence of climatic variability and anthropogenic processes, with increases in cultivated plants corresponding to phases of intensified agricultural activity [33].

These comparisons indicate that the anthropogenic signals observed in Anatolia are not isolated, but form part of a broader pattern of human environment interactions, expressed across multiple spatial scales. This indicates that the spatial variability in human-induced pollen signals in Anatolian lakes is not random but rather arises from the intersection of ecological factors such as altitude, climate, and basin morphology, and socio-economic demands such as proximity to settlement and economic preferences. This suggests that paleoecological interpretations of human-induced pollen signals should be based on region-specific environmental and historical contexts, rather than solely on quantitative thresholds or multiple regional averages.

## 5. Conclusions

This study demonstrates that anthropogenic pollen indicators related to selected BOPs constitute a significant component of pollen-induced vegetation variability in the selected lakes. Therefore, the results should be considered not as a complete reconstruction of anthropogenic influence based on the entire pollen population, but as a comparative assessment of selected anthropogenically derived pollen indicators. The strong correspondence between the anthropogenic indicator taxa and the primary PCA axis indicates that human-induced landscape transformation is embedded within the dominant ecological gradients shaping vegetation dynamics.

The results further reveal marked spatial variability in anthropogenic signals, with some lake records (e.g., İznik and Ova) showing high and dynamic values, while others (e.g., Hoyran and Köyceğiz) exhibit consistently weak signals. This heterogeneity highlights that human impact is not uniformly expressed, but instead reflects locally specific land use strategies, ecological constraints, and socio-economic conditions. The novelty of this study lies not in presenting spatial variability as an entirely unexpected phenomenon, but in comparing the relative prominence and dominant compositional character of anthropogenic pollen indicators within the BOP range in selected lake records from Anatolia. Here, the term relative prominence does not refer to an absolute or independently calibrated measure of human activity intensity. Instead, it refers to a comparison of anthropogenic pollen signals within the dataset based on standardized values of selected anthropogenic indicator taxa. This framework enables direct comparison across sites and facilitates the identification of regionally differentiated patterns of human impact. The results also emphasize the dominant role of Olea europaea as a key proxy of anthropogenic land use, particularly in relation to arboriculture and long-term landscape management. Its strong contribution to the primary PCA axis confirms that tree-crop economies played a central role in structuring vegetation patterns during the Late Holocene. Overall, this study provides a robust and transferable analytical framework for disentangling anthropogenic and climatic drivers in pollen records. Overall, this study provides a robust, semi-quantitative, and transferable analytical framework. It contributes to proxy-based comparative palaeoecological research and offers new insights into the spatial complexity of human–environment interactions in Anatolia during the BOP interval.

## Figures and Tables

**Table 1:** List of pollen records used in this study, including site name, region, Neotoma dataset identifiers, original published sources, and chronology references used for age–depth modelling. The table documents both the primary palynological studies and the chronological frameworks applied to each record, ensuring full transparency, reproducibility, and traceability of the compiled multi-site dataset.

Site	Region	Neotoma Dataset ID	Original Reference	Chronology Source
İznik	NW Anatolia	45170	[16]	[16]
Sapanca	NW Anatolia	45173	[18]	[18]
Abant	NW Anatolia	24161	[17]	[22]
Söğüt	SW Anatolia	4442	[10]	[22]
Beyşehir	SW Anatolia	3927	[10]	[22]
Hoyran	SW Anatolia	24173	[10]	[22]
Gölhisar	SW Anatolia	52268	[13]	[13]
Köyceğiz	SW Anatolia	4161	[10]	[22]
Ova	SW Anatolia	4326	[11]	[22]
Van (VAN1)	Eastern Anatolia	4509	[19]	[22]
Van (VAN2)	Eastern Anatolia	45165	[20]	[20]

**Table 2:** Lake-based summary statistics on the combined pollen percentage of selected anthropogenic indicator taxa in the 1300–3500 cal. BP range.

Lakes	Min (%)	Max (%)	Mean (%)	SD (%)	Total (%)
Abant	0.2	8.1	2.0	2.8	1.7
Beyşehir	0.2	7.0	2.8	2.2	3.0
Gölhisar	0.0	8.6	3.6	2.4	3.9
Hoyran	0.3	2.5	0.9	0.7	0.9
İznik	2.1	36.9	16.6	10.3	16.7
Köyceğiz	0.2	2.9	1.2	0.7	1.3
Ova	0.0	25.4	4.7	7.5	7.9
Sapanca	0.0	4.8	1.5	1.3	1.3
Söğüt	0.7	13.9	4.2	3.3	4.2
Van 1	2.6	7.0	4.5	1.4	4.5
Van 2	2.5	9.0	5.7	1.6	5.5

## Data Availability

The original data presented in this study are openly available in the Neotoma Paleoecology Database, accessible at neotomadb.org.

## Abbreviations

The following abbreviations are used in this manuscript:

BOP	Beyşehir Occupation Phase
PCA	Principal Component Analysis
